# A Multiomic Approach to Investigate the Effects of a Weight Loss Program on the Intestinal Health of Overweight Horses

**DOI:** 10.3389/fvets.2021.668120

**Published:** 2021-06-18

**Authors:** Nicola Walshe, Raul Cabrera-Rubio, Roisin Collins, Antonella Puggioni, Vivian Gath, Fiona Crispie, Paul D. Cotter, Lorraine Brennan, Grace Mulcahy, Vivienne Duggan

**Affiliations:** ^1^School of Veterinary Medicine, University College Dublin, Dublin, Ireland; ^2^Teagasc Food Research Centre, Moorepark, Fermoy, Ireland; ^3^APC Microbiome Ireland, Cork, Ireland; ^4^VistaMilk, Cork, Ireland; ^5^UCD School of Agriculture and Food Science, Conway Institute, UCD Institute of Food and Health, University College Dublin, Dublin, Ireland; ^6^Conway Institute of Biomedical and Biomolecular Research, University College Dublin, Dublin, Ireland

**Keywords:** equine, fecal microbiota, fecal metabolome, multiomics, obesity, weight-loss, diversity

## Abstract

Obesity is endemic in human populations in the western society, and with mounting evidence that the intestinal ecological environment plays a major role in its pathogenesis, identification of therapies based on intestinal microbiota modulation are gaining attention. Obesity in companion animals is also a common clinical problem. We set out using a multidimensional approach, to determine the effectiveness and safety of a weight loss program for horses incorporating diet restriction and exercise. In addition, we aimed to investigate the effect of this program on the overall intestinal health of overweight sedentary horses. The investigation comprised of a randomized, controlled, 6-week study of 14 overweight sedentary horses and ponies who were blocked for age, gender, and breed (controls *n* = 7, treatment *n* = 7). The treatment group were fed a restricted diet (1.4% of body weight dry matter intake) and the control group a maintenance diet (2% of body weight as dry matter intake) over the study period. The treatment group were subjected to a prescribed exercise regime, while the control group were exercised to mimic foraging conditions. Several clinical measurements were taken at the start and end of the study, including morphological parameters, ultrasound measurements of subcutaneous fat, and blood pressure. Fecal microbiota analysis was performed using 16S rRNA gene sequence analysis, and fecal metabolome was analyzed using NMR spectroscopy, on samples taken at weeks 1, 3, and 6 of the study. All horses completed the study period successfully. However, two of the treatment group had to have modified exercise regimes. The treatment group showed significant weight loss (*p* < 0.00001) and an associated decrease in waste circumference (*p* < 0.0001) when compared with the control group. The alpha-diversity of the fecal microbiota in the treatment group showed a significant increase from the start to the end of the study period (*p* < 0.05); however, there was no significant difference between groups at any sampling point. There were significant changes (*p* < 0.05) in the metabolome in both groups between the start and end of the study, but not between groups at any sampling point. Finally, the resting blood pressure of all horses was significantly lower by the end of the study.

## Introduction

Human obesity is recognized as endemic worldwide (1) with comprehensive reports showing a rise in the incidence of obesity globally (2, 3). Domesticated animals also suffer from obesity; notably, 20–51% of selected horse and pony populations are reported to be overweight or obese (4–8).

Obesity is associated with further chronic health problems, including metabolic syndrome. In human metabolic syndrome, obesity and associated increased blood pressure, hyperglycemia, excess abdominal fat, and abnormal blood cholesterol and triglyceride concentrations result in an increased risk of cardiovascular disease and diabetes type-2 (9). Similarly, overweight horses and ponies may develop equine metabolic syndrome (EMS), in which obesity and insulin dysregulation are associated with significant disease (10) such as endocrinopathic laminitis (11, 12), a painful debilitating inflammatory condition of the lamellar tissues connecting the hoof wall to the pedal bone that can ultimately lead to euthanasia (13). Equids with insulin dysregulation are also at risk of dyslipidemia, which may result in hepatic dysfunction (14). In addition, EMS has been associated with myocardial hypertrophy, correlated with insulin dynamics, autonomic nervous system tone, and blood pressure (15).

In order to combat obesity and its associated comorbidities, investigations are ongoing into predisposing factors and potential novel treatments. In humans, this has led to an increasing focus over the past decade on the possible influence of the intestinal microbiota on obesity (reviewed in Castaner et al. (16)). There are a relatively small number of studies investigating the association between the gut microbiota and obese phenotype in the horse; however, changes in the microbiome have been noted, with an increase in microbial diversity in obese horses when compared with a lean cohort (17, 18). Moreover, one study has shown changes in the gut microbiota in horses with EMS; conversely, this study showed a decrease in diversity (19).

The current advice for treatment of EMS is similar to that provided for humans, i.e., diet restriction and controlled exercise (10). There is evidence, indeed, that weight loss through diet restriction and low-intensity exercise can improve insulin sensitivity in obese horses when compared with controls (20). However, there is currently no reported data on the effects of a recommended weight loss program, through diet restriction and exercise, on fecal microbiota or cardiovascular parameters in the horse.

We hypothesized that a weight loss program, through restricted diet and exercise, could not only induce weight loss and improve cardiovascular function in overweight horses, but could also alter the intestinal microbiota to improve gastrointestinal health. The objective of this study was to investigate effects of weight loss on morphological measurements, cardiovascular parameters, fecal microbiota, and fecal metabolome in a cohort of clinically normal but overweight horses and ponies. This was to be achieved using a multiomics approach combined with morphological assessment, cardiovascular diagnostics, and clinical observations.

## Materials and Methods

### Study Population

This was a longitudinal study using client-owned, rather than experimental animals; therefore, the number of animals available were constrained. As we intended to measure a range of parameters related to body condition, physiological parameters, and gut health, we chose to use an anticipated change in body weight as a significant parameter for estimation of required group size.

We estimated that for a study with power = 90%, we could reliably measure an incremental body weight reduction of 5 kg, i.e., a weekly reduction of 1% in an average horse of 500 kg, and that the SD of the reduction would be no more than 4 kg. Hence, the SD units would be 1.25, and the required group size is *n* = 7. Due to the other parameters included in this study and the availability of horses that fit the inclusion criteria, we increased our group size to 14.

The study was carried out on a total of 14 horses. The study population was recruited from a rescue charity (*n* = 12) and from the UCD teaching herd (*n* = 2) that were residents on the study site. The study group consisted of a mix of breeds (Table 1), a range of ages (4–17 years old) and included both genders (nine geldings and five mares). Horses were included in the study if they were considered overweight or obese, i.e., Body Condition Score (BCS) ≥ 6/9 (21), had not received antibiotics or anti-inflammatory treatment in the last 3 months, and had no clinical signs of disease, including clinical laminitis. Animals were excluded from the study if they had any concurrent illness, had a history or evidence of pars pituitary intermedia dysfunction (PPID), or clinical signs of laminitis, i.e., >Obel grade 1 (22). The horses were maintained at UCD Research Farm under the welfare guidelines outlined by UCD Animal Welfare Committee and with exemption from full ethical approval by the Animal Research Ethics Subcommittee (AREC-E-19-26-Mulcahy).

**Table 1:** Outline of study population of 14 horses including horse number, sex, breed, and age.

**Treatment**				**Control**			
**Number**	**Sex**	**Breed**	**Age**	**Number**	**Sex**	**Breed**	**Age**
5	M	Cob pony	5	1	M	Cob	10
7	M	Cob pony	7	2	M	Cob pony	4
8	M	SB	15	3	M	Pony	17
9	M	Pony	5	4	M	SB	5
11	F	Cob	10	13	M	Pony	5
12	F	Cob pony	4	6	F	Cob pony	5
14	F	SB	15	10	F	Cob pony	4

*The treatment group was subject to a weight loss program that involved diet restriction and exercise. The control group remained on a maintenance diet and exercise that mimicked foraging. M, male; F, female; SB, Standard bred*.

### Pre-trial Period

The 12 horses recruited from the rescue charity were treated with anthelmintic (Equest, Pramox 0.4 mg/kg PO, Zoetis Broomhill Rd, Tallaght, Dublin, Ireland) and vaccinated against equine influenza virus and tetanus (Equilis Prequenza TE, MSD Red Oak North, South County Business Park Leopardstown, Dublin 18, Ireland) 2 weeks prior to their arrival on the farm, as per the UCD research farm regulations for any new horses entering the farm.

For the week leading into the trial, the horses were housed on site for acclimatization purposes. The horses were fed 2% of their body weight (BWT) with fresh weight grass hay that was soaked for 1 h prior to administration. They were also made accustomed to an automated horse walker, together with sand paddocks that would be used for turn out purposes during the trial.

### Clinical Monitoring

On arrival, all horses underwent a full clinical examination and lameness examination. Throughout the study, they were monitored daily for food and water intake, fecal consistency, urine output, demeanor, exercise tolerance, and any other observations. They were visually assessed for abnormalities and monitored while exercising on the horse walker for any development of an injury. A trot up examination, which included examination on the turn, was performed weekly to ensure soundness throughout the study. For any horse showing clinical abnormalities, exercise was stopped, and a full clinical examination was performed.

### Study Design

The horses were randomly assigned to control (C) and treatment (T) groups, blocked for age, gender, and breed. The herd remained on site for 45 days incorporating the acclimatization and study periods. The horses were stabled in individual loose boxes on sawdust bedding for the duration of the study. All horses were turned out in pairs for at least 25 min each day, in small sand paddocks to avoid interference with dietary restriction and enable natural social behavior.

### Diet

The C group were kept on a maintenance diet of 2% BWT, as fresh weight hay, which was equivalent to 1.66% dry matter intake (DMI) of pre-soaked grass hay based on a DM of 83%. The T group was restricted to 1.4% BWT as fresh weight grass hay, as per guidelines outlined in the recent EMS consensus statement (10), which is the equivalent of 1.17% DMI of pre-soaked grass hay.

All horses were weighed weekly, and their DMI was adjusted accordingly. The target weight loss for the T group was 1% BWT per week, while the C group was to maintain initial BWT; therefore, the diet was modified weekly in line with weight fluctuations.

The daily DMI was divided into two feeds to be given morning and evening. The hay was placed in slow-feeder hay nets (small gauged hay nets) and weighed individually using hanging scales to calculate DM weight. The filled hay nets were then soaked in individual containers, with weights keeping the nets submerged, for an hour, and allowed to drain for 30 min before feeding. To compensate for loss of nutrients associated with soaking the hay, a multivitamin, mineral, and amino-acid supplement (Equiton^TM^ Troytown Grey-Abbey Pharmacy, Kildare, Ireland) was administered, as per manufacturer's instructions (2 ml/50 kg), in 300 g of high-fiber chaff mix (Hi-Fi lite, Dengie Crops Ltd, Essex, England) once daily and incorporated into the total calculated DMI.

### Exercise

Exercise was carried out on an automated horse walker under continuous supervision with alternating directions every second day. The C group was exercised daily at walk to mimic foraging conditions ensuring that the total time in the walker matched that of the T group. The T group was enrolled in a 5-day weekly walk–trot exercise program, with two rest days. The T group was divided into two subgroups created to accommodate for the length of stride, i.e., horses under 148 cm (T-pony) and horses over 148 cm (T-horse). Exercise intensity was determined by observation, and increases in the exercise program were made in accordance with improved fitness levels and exercise tolerance (Table 2). At every increase in exercise intensity, the horses' heart rates were taken pre-exercise, immediately post-exercise, and 15 min post-exercise to ensure the exercise level was tolerated. On their two rest days, the T group followed the C group horse-walker program.

**Table 2:** Outline of the exercise regime of the treatment (T) group in minutes on automated walker.

**Treatment group**	**Week 1**	**Week 2**	**Week 3**	**Week 4**	**Week 5**	**Week 6**
Warm up	5	5	5	5	5	5
Brisk walk	15	15	15	15	15	15
Trot	0	5	15	15	15	15
Warm down	5	5	5	5	5	5
**Total**	**25**	**30**	**40**	**40**	**40**	**40**

*Level of exercise determined by observation on walker. Exercise was increased from week 1 to 3. Exercise remained at week 3 level until week 6 in line with exercise tolerance of the T group. Bold represent total exercise time for each group*.

### Morphological Parameters

Physical measurements were assessed and recorded before the commencement of treatment and again at the end of the study, as appropriate, by a European Board of Veterinary Specialisation (EBVS) equine internal medicine specialist who was blinded to the group allocation. These included body weight (BWT), body length (BL), height at the withers (HW), body condition score (BCS), cresty neck score (CNS), and measurements of the neck (NC), girth (GC), and waist (WC) circumference. Measurements were taken in triplicate, and the mean value was calculated.

BWT was calculated using an electronic weighing system and was recorded weekly to allow for diet adjustment. BL was calculated from the point of the shoulder to the point of the ischium using a soft tape with 1-cm increments. HW was calculated using a wooden horse measuring stick. BCS was calculated by visual assessment and palpation using a nine-point scale, i.e., 1 (poor) to 9 (extremely fat) (21), and the CNS was calculated using a six-point scale, i.e., 0 (no visual appearance of a crest) to 5 (crest is so large, it permanently droops to one side) (23). NC was calculated at the upper third, middle third, and lower third of the neck, and an average of those three measurements was also recorded. GC was measured around the thorax behind the withers, and WC was measured at the widest point of the abdomen.

Resting blood pressure (BP) was measured non-invasively using a portable veterinary blood pressure monitor (Cardell Veterinary Monitor 9401; Sharn Veterinary Inc., Florida, USA). SV 8 (9 cm) and SV 10 (12 cm) cuffs were used, as appropriate for the size of the horse/pony to provide a snug fit with the hook and loop sections fully engaged. The cuff was applied to the base of the tail in the standing horse or pony to approximate the level of the right atrium. Mean blood pressure measurement was repeated five times at each recording time, and the average value was calculated. Clear outliers were discarded.

### Ultrasound of Subcutaneous Fat

Subcutaneous fat thickness (SFT) measurements were made by a European College of Veterinary Diagnostic Imaging (ECVDI®) specialist who was blinded to the group allocation. If the horse's demeanor allowed, consistent with handler safety, the area was clipped, sprayed with 70% alcohol solution, and transmission gel applied. When clipping was not possible, large amounts of the alcohol solution were used to obtain images of the diagnostic value. The transcutaneous B-mode ultrasound examinations were performed using a GE Logiq e ultrasound machine (IVM imaging Ireland, Gormanston, Co. Meath), equipped with a linear probe (12L-RS 5–13 MHz). A technique described in a previous study (24) was modified, and five areas were examined for subcutaneous fat measurements; these included retroperitoneal (ventral midline), top of the crest of the neck, withers, ribs at the 14^th^ intercostal space (ICS), and the tailhead. From the right side of the horse, images for each area were obtained in triplicate and saved; SFT was then measured and calculated to the nearest 1 mm. The average of the measurement in three images per location was used for statistical analysis. All measurements were taken before the commencement and after the cessation of the weight loss program.

### Fecal Sample Collection

Fecal samples were collected as per recommendations for microbiota analysis outlined by (25) on the same day at the same time each week. The time point of sampling was attributed in accordance with week number of the study, i.e., week 1 was time point one (TP1), week 3 was timepoint 3 (TP3), and week 6 was time point six (TP6). Freshly voided feces that had been produced after the horses' morning feed were used. The fecal ball that was least contaminated with bedding was selected and placed in a sterile container, which was sealed, labeled, and immediately put on ice.

### Initial Processing of Fecal Samples

Initial processing of samples was carried out within 6 h of collection. The fecal ball was removed from the container, and a 10-g sample was taken from the center of the ball (25) for both metabolome and microbiota analysis and placed in a sterile container. These samples were numerically labeled so as not to bias the analysis and were stored at −80 °C until further processing.

### Fecal Sample Preparation and 16S rRNA Gene Sequence Analysis

Fecal samples for microbiota analysis were processed as described previously (26). Briefly, samples were homogenized and processed using mechanical and chemical lysis. DNA was extracted using the QIAamp® PowerFecal® Pro DNA Kit (QIAGEN®). DNA concentration was normalized, and 16S libraries were prepared using primers to amplify the V3–V4 region of the bacterial 16S rRNA gene, with Illumina adaptors incorporated as described in the Illumina 16S Metagenomic Library Preparation guide. Following index PCR and purification, the products were quantified using the Qubit high-sensitivity DNA kit (Life Technologies) and pooled equimolarly. The pooled libraries were assessed using an Agilent high-sensitivity DNA kit and quantified by quantitative PCR (qPCR) using the Kapa Quantification kit for Illumina (Kapa Biosystems, USA) according to the manufacturer's guidelines. Libraries were then diluted and denatured following the Illumina platform guidelines and sequenced (2 × 300 bp) on the Illumina MiSeq platform.

The sequences obtained were filtered on the basis of quality (removal of low-quality nucleotides at the 3′ end and removal of window 10 nt with low average quality) and length [removal of sequences with <200 bp with prinseq as per the Schmider and Edwards protocol (27), and the paired-end reads with a minimum overlap of 50 bp were joined using Fastq-join (28)]. Finally, all single files were processed to a final filtering sequence mean quality score >25. The filtered sequences were matched at operational taxonomic unit 241 (OTU; with 97% identity level) using the UPARSE-OTU algorithm with usearch v7.0 program (29), and Chimeric sequences were removed with ChimeraSlayer with the Gold database (https://gold.jgi.doe.gov) and UCHIME. The taxonomic assignment of these OTUs was matched to results in the Ribosomal Database Project (30) and were labeled unclassified if the bootstrap value was less than 80%. Alpha and beta diversities were calculated using phyloseq and Vegan R packages (31). The R MixOmics package (32) was used to carry out multiomic analysis, to enable the use of relevant network options to visualize the relationship between the selected variables and results of each group and time point.

### Fecal Metabolome Analysis

Fecal water was extracted from fecal samples as previously described (33). To prepare the samples for NMR spectroscopy, 60 μl of D_2_O and 10 μl of sodium trimethylsilyl propionate-[2,2,3,3-2H4] (TSP) (0.05 g/ml) were added to 540 μl of fecal water. The NMR spectra were acquired on a 600-MHz Varian NMR spectrometer by using a CPMG pulse sequence at 25°C. The spectra were acquired with 16,384 data points and 128 scans. Water suppression was achieved during the relaxation delay of 3 s. All ^1^H NMR spectra were referenced to TSP at 0.0 parts per million (ppm) and processed manually with the Chenomx NMR Suite (version 7.5) by using a line broadening of 0.2 Hz, followed by phase correction and baseline correction. The spectra were integrated into regions in domains of 0.001 ppm excluding both TSP and water regions. Data were normalized to the sum of the spectral integral. Metabolites were identified using Chenomx NMR Suite (version 7.5) and the HMDB database.

### Statistical Methods

The SPSS statistical software was used to estimate normality using the Shapiro–Wilk test. Two-tailed Student *T*-tests were used to compare changes in physical parameters that were normally distributed. The Mann–Whitney U test was used for non-parametric data, i.e., BCS and CNS.

Metabolome data analysis was performed using both SIMCA (multivariate) and MetaboAnalyst (https://www.metaboanalyst.ca/) (univariate). PCA analysis was performed using SIMCA, and the data were par scaled before analysis. Two-way repeated measures ANOVA was performed using MetaboAnalyst, and the data were corrected for multiple comparisons using the false discovery rate (FDR) procedure in MetaboAnalyst.

Microbiota analysis was performed using downstream analyses, and graphical outputs of the 16S rRNA results were generated with various packages in R (31). For beta-diversity analysis, the dissimilarity matrix between samples was calculated with the Bray–Curtis method transforming the data into a logarithmic scale, studying the effects on microbiota composition. The variability between samples by permutational multivariate analysis of variance was calculated using distance matrices from the R Vegan package (34). For alpha-diversity values, assumption of normality was checked using the Shapiro–Wilk test. Potential differences in alpha-diversity included in the study were thereafter estimated by repeated measures analysis of variance (ANOVA) and *t*-tests. Linear discriminant analysis effect size (LEfSe) was performed in order to discover specific bacterial biomarkers associated with control and treatment groups. Statistical differences between multiple samples at the phylum, family, and genera levels were determined by both Kruskal–Wallis and Mann–Whitney U-tests, adjusting for multiple testing (35) with the R statistical package (https://www.r-project.org/). Statistical significance was established at *p* < 0.05.

## Results

### Study Population and Clinical Monitoring

All 14 horses recruited to the study, completed the trial.

Over the 6-week period, there were four horses, Horse 1 (C group) and Horses 7, 11, 13 (T group), that presented with signs of clinical disease. These individuals were treated by changes in environmental management, dietary management, or exercise regime (see Table 3 for details). No chemical therapeutics were used for any of the 14 horses at any stage.

**Table 3:** A summary of the horses who presented with signs of clinical disease during the 6-week trial period.

**Number**	**Group**	**Week**	**Presentation**	**Treatment**
1	Control	1	Increased respiratory effort at rest	Moved to outdoor stable
7	Treatment	2	Acute moderate weight loss, lethargy, and transient soft but formed fecal consistency	Increased feed intake to 2% BWT DMI
11	Treatment	1	2/5 grade (AAEP) lameness right fore associated with poor foot balance	Decreased level of exercise to control group level
12	Treatment	3	3/5 grade (AAEP) lameness right fore with tissue swelling and heat associated with the medial mid cannon area	Rehabilitation program combined with daily local hydrotherapy

*The table outlines the horse affected, the presenting complaint, and the treatment*.

### Diet

DMI was adjusted weekly in both groups. This ensured that there was no change in BWT of the control group and that the target of 1% BWT loss was obtained weekly in the treatment group. Therefore, the range of restriction on DMI in the treatment group ranged from 1.3 to 2% BWT DMI in accordance with weight loss or gain. Hay from a second source was introduced incrementally into the diet starting on day 18 with the inclusion of 10% of the “new” hay in the total DMI, increased by 10% increments every 2 days so as to reduce the risk of intestinal upset.

### Exercise

In the treatment group, five of the seven horses participated in the prescriptive exercise program. As mentioned above, two horses sustained injuries and, thus, had modified exercise regimes. Horse 11 joined the control exercise program. Horse 12 was enrolled in a rehabilitation program that began as 10 min walking in hand twice daily on day 15 with a gradual increase in time to include trotting for 10 min twice daily by day 25 (Table 3).

### Morphological Parameters

There was a significant loss of BWT in the treatment group (*p* < 0.00001) compared with the control group (Table 4). The treatment group lost, on average, 1% BWT a week, with an average of 4% BWT lost over the duration of the study. There was also a significant decrease in the WC in the treatment group (*p* < 0.0001) compared with the control group, with an average decrease in circumference of 3% (Table 3). Resting blood pressure was significantly lower in all horses and ponies at the end of the study, compared with the start (*p* = 0.0007) with no significant difference between the treatment and control groups.

**Table 4:** A summary of the significant morphological parameter findings between week 1 and week 6 of the study.

**Horse**	**BWT**			**WC**			**BP**		
	**Start**	**End**	**Loss/gain**	**Start**	**End**	**Loss/gain**	**Start**	**End**	**Loss/gain**
	**kg**	**kg**	**%**	**cm**	**cm**	**%**	**mmHg**	**mmHg**	**%**
**Control**									
1	640	635	−0.8%	226.33	226.67	0.15%	92.2	86.4	−6.29%
2	378	382	1.1%	182.67	186.33	0.02	111	82	−26.13%
3	320	320	0.0%	189.67	196.00	3.34%	112.6	86.4	−23.27%
4	496	499	0.6%	198.00	205.33	3.70%	NT	NT	NT
6	568	576	1.4%	215.33	222.00	3.10%	99.4	87.8	−11.76%
10	408	408	0.0%	194.00	197.00	1.55%	108.4	85.2	−21.04%
13	372	373	0.3%	189.33	192.67	1.76%	91.2	91	−0.22%
**Treatment**									
5	453	430	−5.1%	187.67	183.67	−2.13%	116.4	90.6	−22.16%
7	358	338	−5.6%	192.67	185.00	−3.98%	116.8	56.2	51.88%
8	528	511	−3.2%	209.67	209.33	−0.16%	107.4	75	−30.17%
9	260	249	−4.2%	171.33	165.33	−3.50%	92.2	85.8	−6.94%
11	656	633	−3.5%	228.00	225.00	−1.32%	130	117	−10.31%
12	484	470	−2.9%	209.00	202.33	−3.19%	109.2	91.6	−16.12%
14	608	586	−3.6%	237.67	226.00	−4.91%	82.8	67.2	18.84%

*The treatment group was subject to a weight loss program that involved diet restriction and exercise. The control group remained on a maintenance diet and light exercise to mimic natural foraging. BWT, body weight; WC, waist circumference; BP, blood pressure; NT, not taken*.

### Ultrasound of Subcutaneous Fat

There were no significant differences found between week 1 and 6 in subcutaneous fat measurements at any location in either treatment or control group (Supplementary Table 1).

### Fecal Sample Collection

Fecal samples were collected in weeks 1, 3, and 6 at the same time on the same day (Wednesday between 8 a.m. and 10:30 a.m.), with the exception of one sample from horse 9 in week 1, which was collected at 4 p.m. on Wednesday afternoon in week 1.

### Fecal Microbiota Analysis

**Figure 1:**
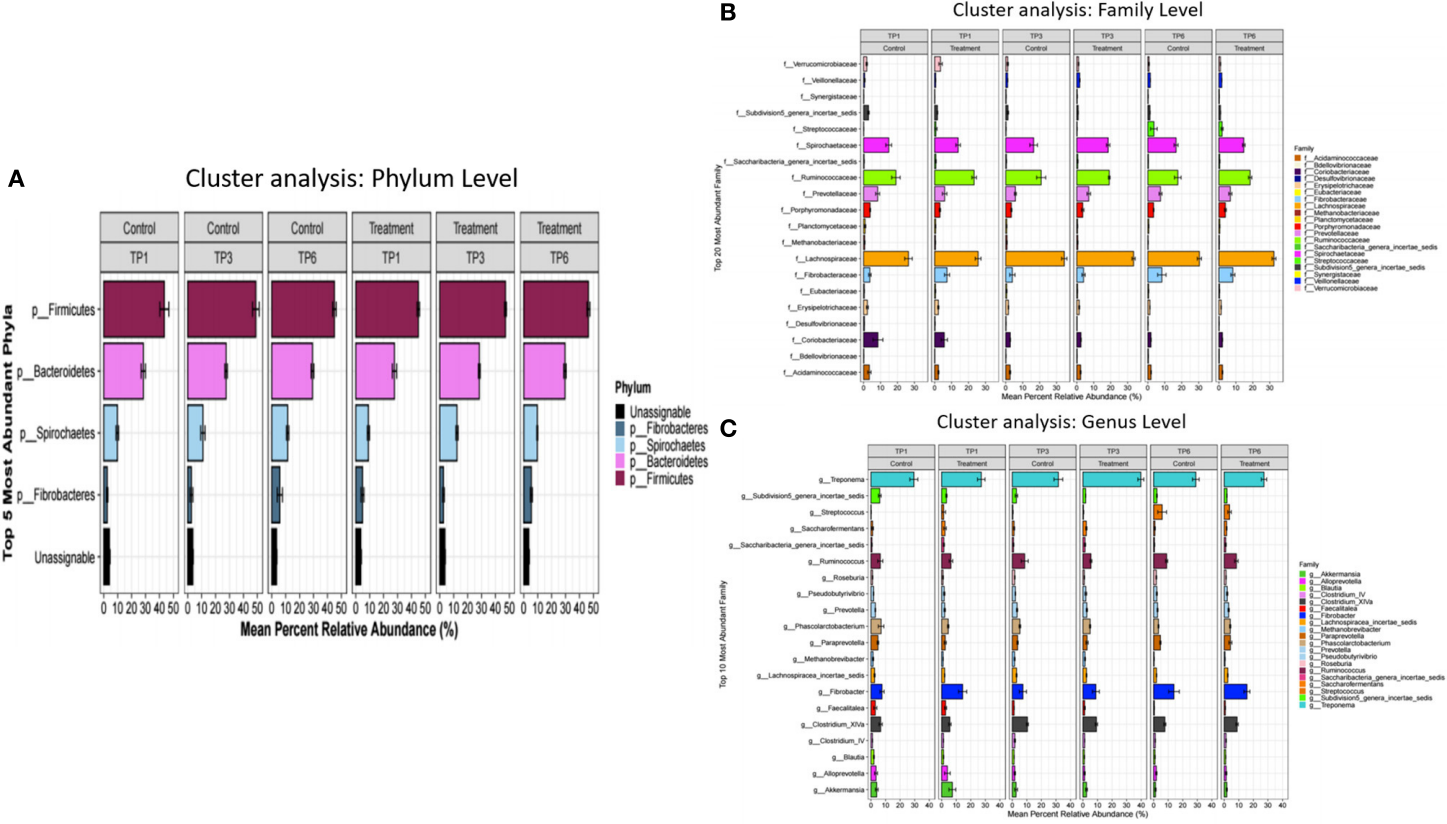
The mean percentage relative abundance of the major phyla **(A)**, families **(B)**, and genera **(C)** in the control and treatment groups over three timepoints. The treatment group (*n* = 7) was subject to a weight loss program that involved diet restriction and exercise. The control group (*n* = 7) remained on a maintenance diet and light exercise to mimic natural foraging. TP, time point; TP1, week 1; TP3, week 3; TP6, week 6.

The analysis of the fecal microbiota showed general similarities at the phylum, family and genus level between the treatment and control groups across all time points (Figure 1). Alpha diversity showed no significant differences between the T and C groups when compared with each other at the specific three sampling time points.

**Figure 2:**
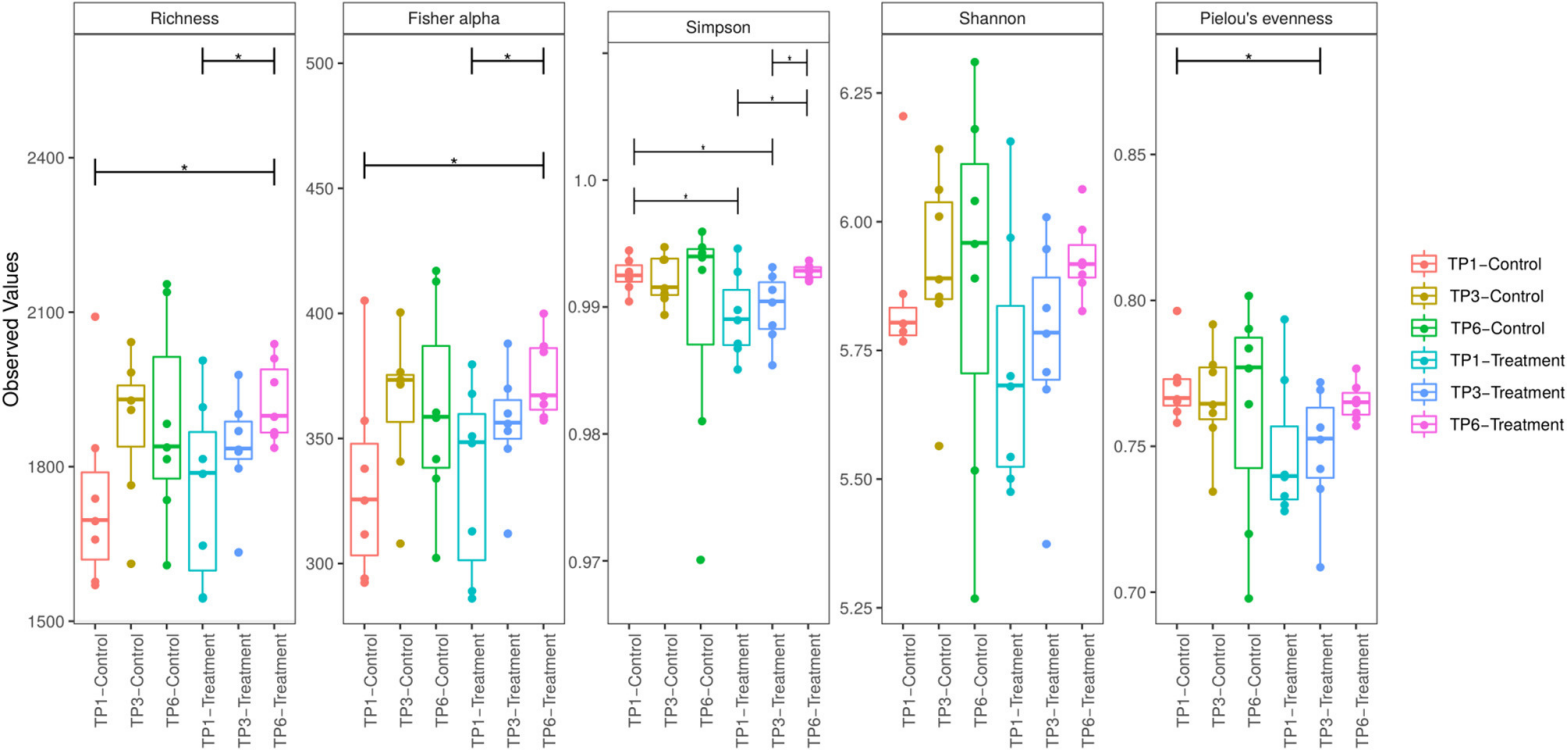
Alpha diversity was measured by five indices of diversity: Richness, Fisher Alpha, Simpson, Shannon, and Pielou Evenness. The treatment group (*n* = 7) was subject to a weight loss program that involved diet restriction and exercise. The control group (*n* = 7) remained on a maintenance diet and light exercise to mimic natural foraging. The group and timepoint are color coded. The T group showed a significant increase in diversity between timepoint 1 and timepoint 6 as measure by Richness, Fisher alpha, and Simpson indices. TP, timepoint; TP1, week 1; TP3, week 3; TP6, week 6. *p < 0.05.

However, when comparing diversity within groups over time, a significant increase in alpha diversity, Fisher Alpha index, and Richness index, between TP1 and TP6 in the T group (Figure 2) was identified, while a trend toward increased Shannon diversity was apparent. Similarly, evenness was significantly increased (Simpson). There were no differences in the alpha diversity of the C group between time points (Figure 2).

**Figure 3:**
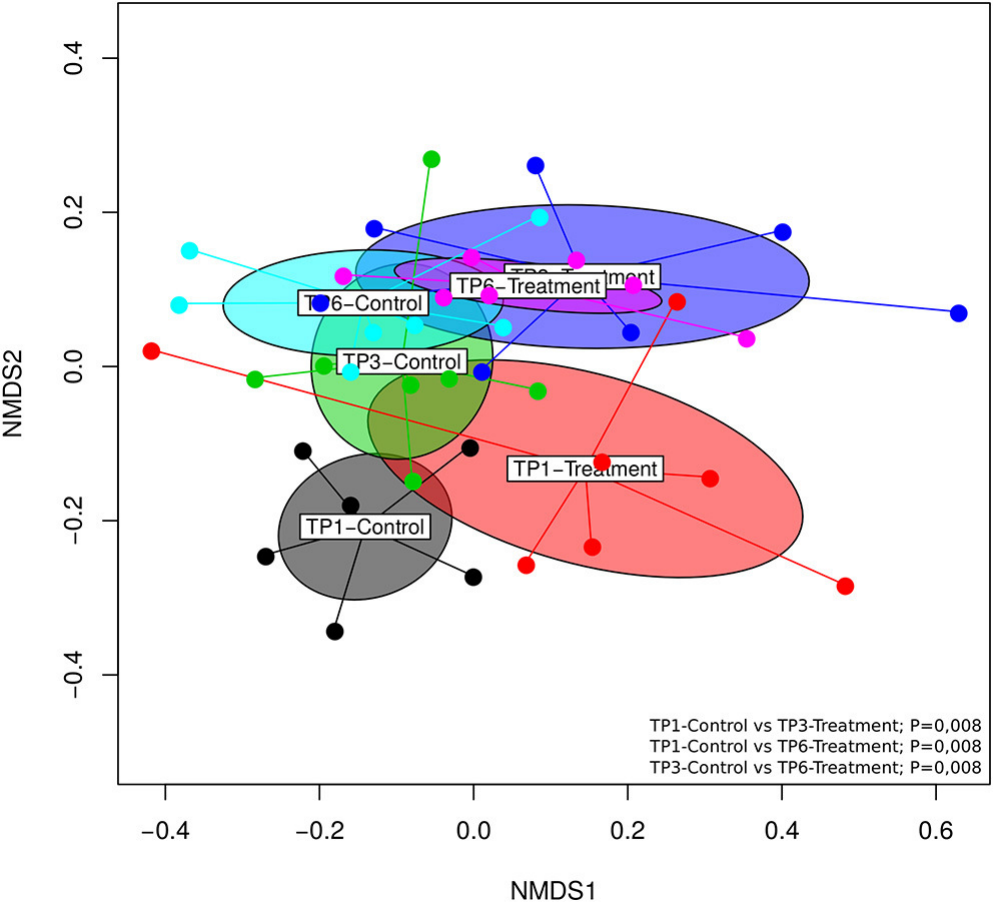
The beta diversity illustrated using an NMDS graph; the treatment and control groups are shown at each timepoint and are color coded. The treatment group (*n* = 7) was subject to a weight loss program that involved diet restriction and exercise. The control group (*n* = 7) remained on a maintenance diet and light exercise to mimic natural foraging. The significant differences found included: TP1-control vs. TP3-treatment (*p* < 0.008), TP1-control vs. TP6-treatment (*p* < 0.008), and TP3-control vs. TP6-treatment (*p* < 0.008). TP, time point; TP1, week 1; TP3, week 3; TP6, week 6.

Significant differences (*p* = 0.001) in beta diversity (NMDS, Bray–Curtis) were evident (Figure 3). Pairwise comparison of NMDS distances revealed significant differences between TP1-Control vs. TP3-Treatment (*p* < 0.008), TP1-Control vs. TP6-Treatment (*p* < 0.008), and TP3-Control vs. TP6-Treatment (*p* < 0.008) (Figure 3). Also, there was evidence of clustering according to individual animal in the treatment group (Supplementary Figure 1).

**Figure 4:**
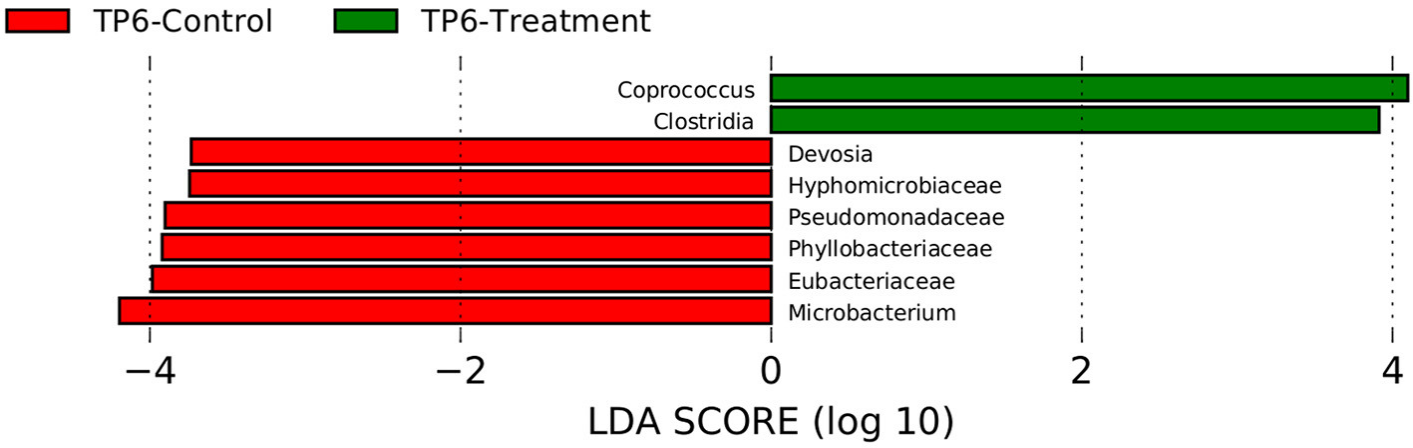
Linear discriminant analysis effect size (LEfSe) was performed in order to discover specific bacterial biomarkers associated with control and treatment groups. The treatment group (*n* = 7) was subject to a weight loss program that involved diet restriction and exercise. The control group (*n* = 7) remained on a maintenance diet and light exercise to mimic natural foraging. In this graph, statistically significant differences of samples between the T and C groups at timepoint 6 are shown. Significance (*p* < 0.05). TP6, timepoint 6.

Assessment with linear discrimination analysis (LDA) effect size >3 and LefSe (Wilcoxon test) showed statistically significant differences when respective groups at specific time points were directly compared. More specifically, there was a significant reduction in the relative abundances of the families Eubacteriaceae (*p* < 0.05) and Pseudomonadaceae (*p* < 0.05), as well as an increase in the genus *Coprococcus* and the class Clostridia in the T group compared with the C group at timepoint 6 (Figure 4).

### Fecal Metabolome Analysis

The NMR metabolomics data was successfully acquired from all fecal samples at each sampling timepoint. A representative NMR spectrum is displayed in Supplementary Figure 2.

**Figure 5:**
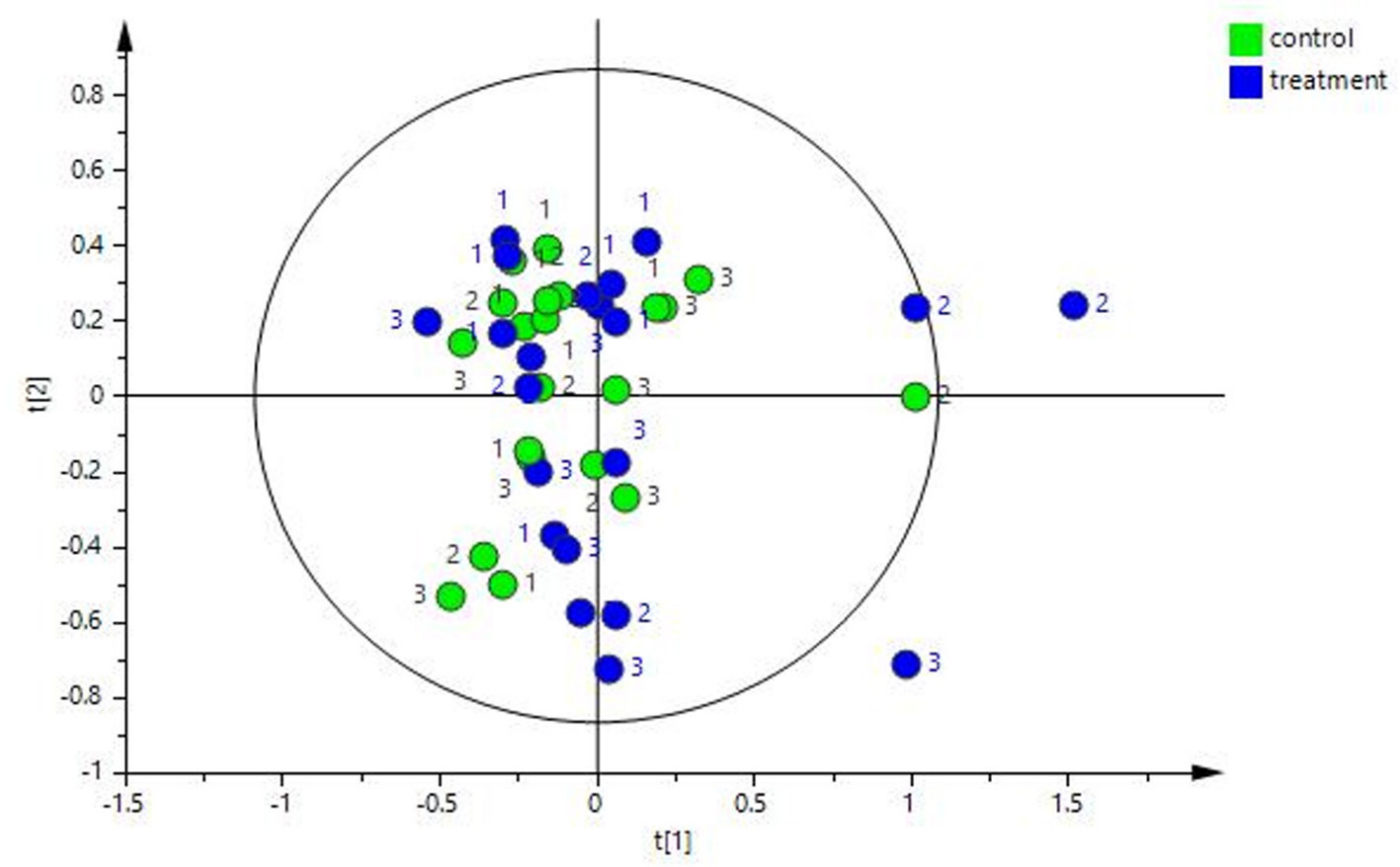
PCA plot of the fecal water NMR data color coded according to group (treatment and control). The treatment group (*n* = 7) was subject to a weight loss program that involved diet restriction and exercise. The control group (*n* = 7) remained on a maintenance diet and light exercise to mimic natural foraging. No significance difference on the global metabolic profile was found between groups.

The multivariate analysis revealed that there was no major impact on the overall fecal metabolomic profile that could be attributed to treatment, i.e., the exercise and diet program (see Figure 5, R2 0.89). However, there was a significant change in fecal metabolome composition over time (i.e., from T1 to T6) in both the control and treatment group but no significant differences between the groups at any timepoint.

**Figure 6:**
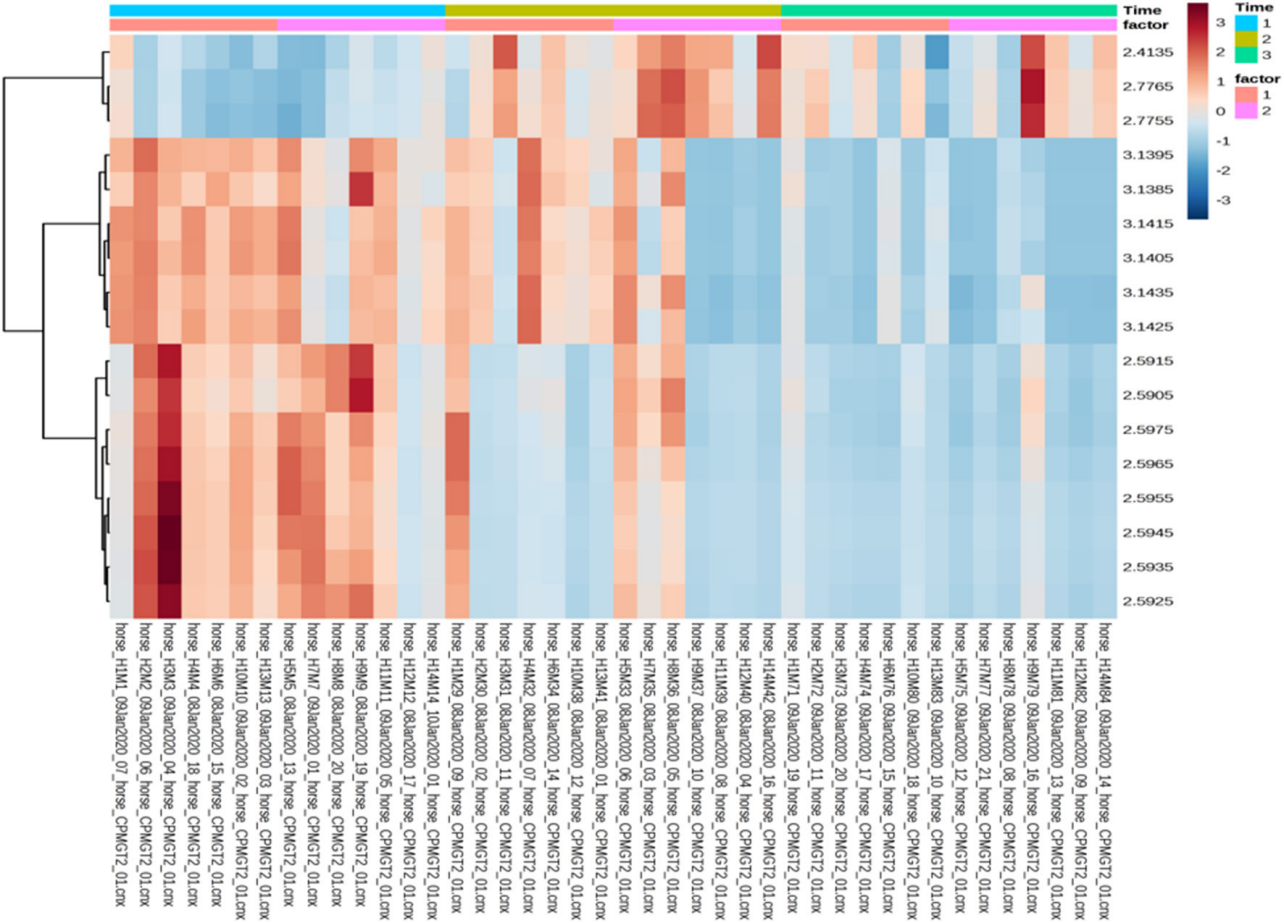
Heatmap analysis of the treatment and control groups at three timepoints during the study. The treatment group (*n* = 7) was subject to a weight loss program that involved diet restriction and exercise. The control group (*n* = 7) remained on a maintenance diet and light exercise to mimic natural foraging. The analysis of the significant features shows the changes in treatment group/control group across the all the timepoints, with no significance seen between groups at any timepoint. Time 1, 2, and 3: TP1, week 1; TP3, week 3; TP6, week 6; Factor 1, control; Factor 2, treatment; Y axis, metabolites; X-axis, horse and sample number.

Univariate data revealed changes in the metabolome profile over the study period irrespective of group, and a wo-way ANOVA analysis revealed that there were specific metabolites (dimethylsulfone and methylamine) with a significant factor effect (control/treatment) (Table 5). There was a decrease in the levels of dimethysulfone and methylamine metabolites, and there was an increase in unknown metabolites (2.4135, 2.7755, 2.7765) in both groups from TP1 to TP6. This is demonstrated in the heatmap analysis of the significant features in each group over the study period (Figure 6).

**Table 5:** A two-way ANOVA analysis of metabolites taking into account the sample timepoint (Time) and the effect of the group (Factor), i.e., control vs. treatment group.

**NNMR data**		**Time(raw.p)**	**Time(adj.p)**	**Factor(raw.p)**	**Factor(adj.p)**	**Interaction(raw.p)**
3.1405	Dimethylsulfone	0.006449	0.41649	2.39E−07	0.000835	0.30133
3.1415	Dimethylsulfone	0.008492	0.41649	9.72E−07	0.001501	0.39066
3.1395	Dimethylsulfone	0.010088	0.41649	1.29E−06	0.001501	0.42122
3.1425	Dimethylsulfone	0.011623	0.41649	8.11E−06	0.006362	0.54783
3.1435	Dimethylsulfone	0.019075	0.41649	6.61E−05	0.017793	0.69828
3.1385	Dimethylsulfone	0.048665	0.41649	4.58E−05	0.014563	0.54441
2.7765	Unknown	0.05342	0.41649	0.000239	0.049286	0.44754
2.7755	Unknown	0.058283	0.41649	0.000185	0.04324	0.49582
2.4135	Unknown	0.16904	0.41649	0.00022	0.048163	0.37797
2.5945	Methylamine	0.36387	0.52756	1.70E−05	0.008492	0.57962
2.5955	Methylamine	0.39142	0.54966	2.39E−05	0.009409	0.64331
2.5935	Methylamine	0.47239	0.61876	1.09E−05	0.006362	0.64923
2.5965	Methylamine	0.51512	0.65026	3.00E−05	0.010497	0.74757
2.5905	Methylamine	0.64991	0.74924	0.000141	0.035276	0.75996
2.5975	Methylamine	0.72159	0.7947	5.70E−05	0.016639	0.78131
2.5925	Methylamine	0.73149	0.79781	9.65E−06	0.006362	0.8052
2.5915	Methylamine	0.90805	0.92766	2.42E−05	0.009409	0.83179

*Note. Metabolites that had a significant factor effect (control/treatment) are listed. No difference was attributed to Group at any timepoint*.

### Multiomic Analysis

The relationship between microbiota and metabolites in the feces, in the control and treatment groups over the three different time points, was visualized through Pearson network images of correlation. The total NMR profile and OTU readings for each sample was used in this analysis, to identify any significant correlations between fecal microbiota and metabolites in both groups at each time point. A correlation analysis cutoff was used (*r* = 0.8), with the color of the lines between the nodes in each case indicating the strength and direction (positive/negative) of the correlation.

**Figure 7:**
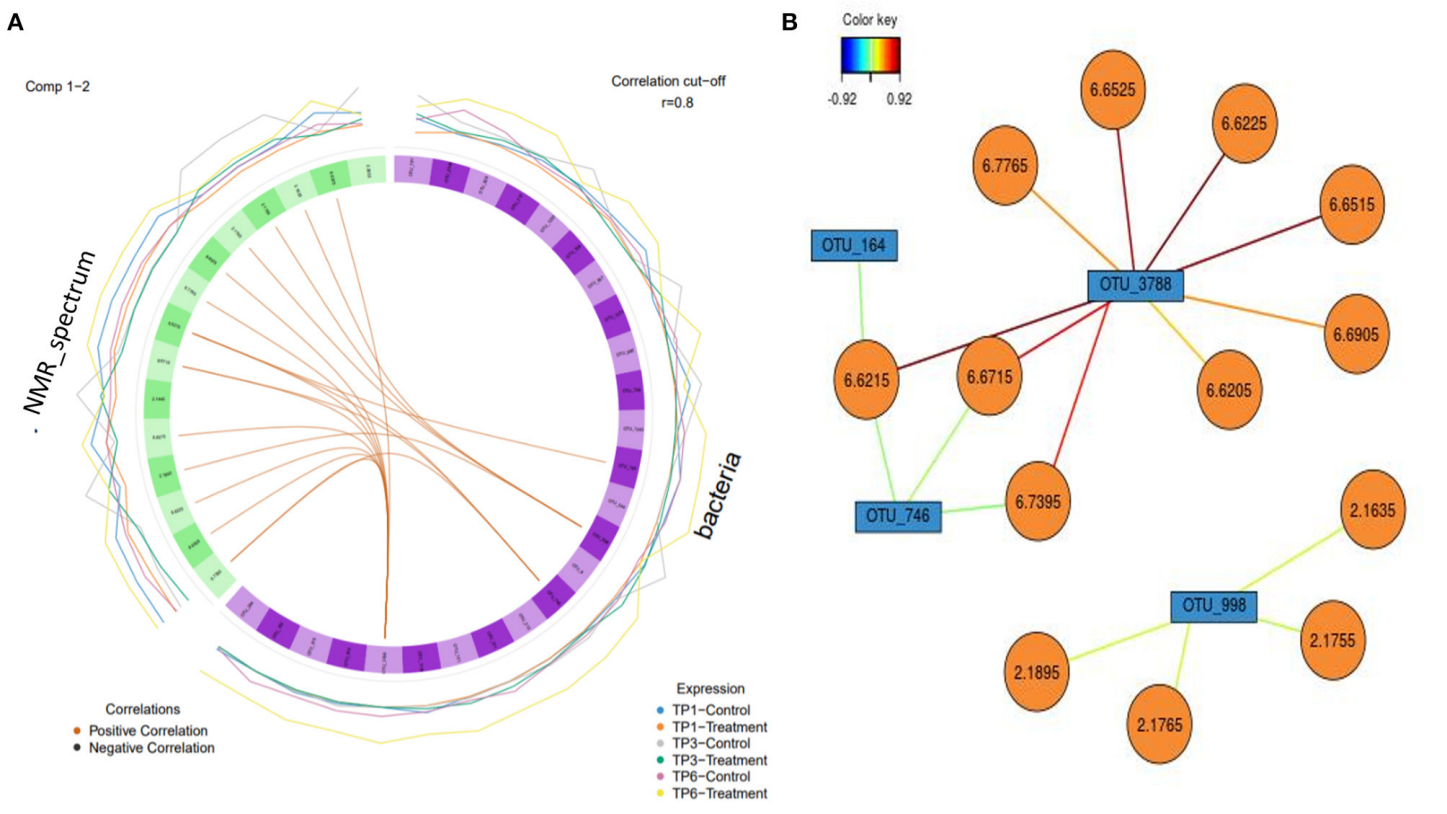
**(A)** A circos plot of the Spearman correlation between “bacteria,” i.e., operational taxonomic units (OTUs) (in purple) and “NMR spectrum,” i.e., metabolites (in green). Each individual feature is labeled within the block. The outer lines indicate the expression level of each variable according to group over each timepoint, e.g., blue is the control group over timepoint 1. The correlation is colored coded to show positive and negative as orange and black, respectively (*r* = 0.8). The treatment group (*n* = 7) was subject to a weight loss program that involved diet restriction and exercise. The control group (*n* = 7) remained on a maintenance diet and light exercise to mimic natural foraging. TP1, week 1; TP3, week 3; TP6, week 6. **(B)** Relevance network with each color representing a significant variable that was highlighted in the circos plot and the associated correlation (*r* = 0.8) with the strength of correlation color coded. OTUs are in blue, and the metabolites are in orange.

A circos plot with a correlation cutoff of *r* = 0.8 (Figure 7) shows that OTU 3778 (p__Bacteroidetes; c__Bacteroidia; o__Bacteroidales; f__Rikenellaceae) is strongly associated with the NMR peaks corresponding to urocanic acid (6.6225, 6.6215, 6.6525, and 6.6515 ppm), with additional peaks of 6.7765, 6.6205, and 6.6905 (unidentifiable metabolites) associated with the same OTU (3,788). This analysis also shows propionate (2.1635, 2.1895, 2.1765, and 2.1755 ppm) is associated with OTU 998 (p__Firmicutes; c__Clostridia; o__Clostridiales; f__Ruminococcaceae). OTU 164 (Firmicutes; c__Negativicutes; o__Selenomonadales; f__Acidaminococcaceae; g__Phascolarctobacterium) and OTU 748 (p__Firmicutes; c__Clostridia; o__Clostridiales; f__Ruminococcaceae) weakly correlated with urocanic acid.

The significant correlations between OTU 3778 and associated metabolites were only found in the T group at TP 3. The correlation between the other three OTUs (OTU 998, OTU 164, and OTU 748) was found at all timepoints in both groups; however, the association was more evident in the T group.

## Discussion

Obesity is endemic in human populations in the developed world, and this has spread into our domesticated animal population with reports of over 50% of the pet population being overweight or obese (4, 5, 36). The concept of obesity goes back to ancient times (37), yet scientists are still grappling to instigate successful interventions that can effectively combat the rising tide of obesity and associated diseases. This has prompted investigations into the merits of manipulating the intestinal microbiota in order to ameliorate obesity-associated gut dysbiosis. In line with this, this study investigated the effects of a weight loss program through exercise and diet restriction in horses on the associated intestinal gut microbiota and cardiovascular parameters. This program was successful in reducing weight and waist circumference, and there was an associated increase in microbial diversity between the start and end of the study period in the treatment group. Both treatment and control groups had lowered blood pressure at the end of the study, likely as a result of environmental adaptation and frequent handling, and there were adverse effects on the gastrointestinal system in either group.

### Weight Loss

Dietary restriction and controlled exercise are the foundations of treatment of obesity in both humans and horses. In this study, significant weight loss was achieved in a group of overweight horses. This is in line with similar studies that implemented both diet and exercise as part of a weight loss program (20, 38–40). This study also recorded a significant decrease in WC, similar to that previously observed in a diet restriction program in horses with EMS (41). In several previous studies, exercise or exercise combined with diet restriction was associated with increased insulin sensitivity (38, 39, 41–43). Insulin dysregulation has been shown to affect the gut microbiota of ponies (17) and horses suffering from EMS (19). Although there were varying and few significant differences between the fecal microbiota in the weight loss and control groups in this study, further investigation of these changes along with the dynamic link between insulin regulation and the gut microbial environment would be of value.

### Morphological Parameters/US

Although significant weight loss and decreased WC were recorded in the treatment group, these changes were not reflected in the remaining morphological parameters measured including BCS or the subcutaneous fat ultrasound measurements. This is consistent with another study of 12 overweight/obese horses that were submitted to exercise-induced weight loss but had no change in subcutaneous fat measurements (44) and supported by the long periods taken to change subcutaneous fat levels significantly as measured by ultrasound analysis (24, 45). A previous diet restriction study in horses with EMS did show a similar reduction in WC to that observed in the current study, with a corresponding decrease in BCS (41). This could be due to a decrease in internal abdominal fat or perhaps a toning of the abdominal muscles due to increased levels of exercise. A decrease in BCS associated with weight loss has also been seen in a number of studies (20, 42, 43, 46), with a decrease in NC associated with exercise and the corresponding insulin sensitivity in one of those (43). Therefore, it appears that BWT loss, WC, and BCS remain the most reliable reflections of weight loss in the horse and that changes in other parameters may require a program of longer duration or a more restricted diet.

### Cardiovascular

It has been shown in both human (47) and equine studies (15) that improved body composition was associated with improved cardiovascular function, and it has been proposed that cardiovascular disease could be prevented by exercise-induced changes to the intestinal microbiota (48). In this study, improvements in the cardiovascular parameter of resting blood pressure was seen in both treatment and control groups; however, we speculate this was due to a controlled level of exercise for all groups regardless of intensity. Habituation to frequent handling may also have influenced resting blood pressure.

### Diet Restriction

Although the recommendations for dietary restriction in overweight horses is 1.4% BWT (10), it has been shown that restrictions using 1% of BWT in DMI can be used successfully without any detrimental effects to the horses' welfare (49), and in some weight-loss resistant animals, this level of restriction is necessary (50). In this study, we found individual variation of weight loss or gain, which needed to be monitored strictly to achieve the recommended loss of 1% BWT per week (treatment group) (10) or to keep the horses at their presented weight (control group). Therefore, regardless of level of restriction, consistent monitoring of weight to allow for dietary adjustment should be a key part of any weight loss program, as some horses may be more weight loss resistant than others (50).

### Fecal Multiomic Analysis

Due to the complexity of bacterial and host interactions, this study used a multiomic approach broadening the investigation to incorporate not only the structure of the microbial community but also the metabolic functions. This study analyzed the microbiota and metabolites found in the feces of overweight, sedentary horses undergoing a strict weight loss program compared with a control cohort maintained at their presenting weight.

There are a few studies investigating the effect of obese phenotype on the intestinal environment in horses, often using one sample point and a heterogenous population (18, 51), or sampling only at the start and end points of a homogenous population, diet controlled, uniform location studies (17, 52). As described, this is a longitudinal study with three sample points during the weight loss phase allowing for analysis of changes over time as recommended by Dougal (53).

The composition of the major phyla found in equine feces in this study is consistent with other studies, dominated by Firmicutes, Bacteroidetes, and then the less dominant phyla of Spirochaetes and Fibrobacteres (54–57). At each sample point, Firmicutes was the predominant phylum over Bacteroidetes in these overweight horses. A decrease in Bacteroidetes was associated with obesity in humans (58); however, this finding was contradicted in a later study with a larger study group size (59) and Firmicutes: Bacteroidetes ratio is no longer regarded as a reliable metric. In horses, a predominance of Firmicutes was found in obese horses using one sampling point (*n* = 37) (18), but the opposite was found in a study of obese Welsh mountain ponies (52) indicating that reports of the importance of phylum-level changes with respect to obesity should also be interpreted with extreme caution in the equine setting.

The taxonomic composition at the family and genus levels were broadly the same across the most abundant bacteria (Figure 1). However, the LEFSE analysis of the C- and T-groups at TP6 did show significant differences. Notably, there was an increase in the class Clostridia and the genus *Coprococcus* in the T group at the end of the weight loss program. The class Clostridia has been shown to be altered in obesity in many rodent data sets, with increased abundance more commonly associated with obese state (60). However, within such a broad-ranging class, orders such as Clostridiales have been associated with intestinal health particularly in relation to exercise (61, 62), and a decreased abundance has been associated with colitis (63) and colic (64) in horses. Also, families such as Ruminococcaceae and Lachnospiraceae have been associated with normal horses when compared with horses with EMS (19). *Coprococcus* abundance has previously been negatively correlated with body mass index and body fat percentage (65) and to be decreased in obese adults (66), patterns that are consistent with our findings. However, this genus has also been found to be highly enriched in young obese individuals (67) and associated with obesity and metabolic syndrome in women (68).

It was notable that there was an increased relative abundance of the families Eubacteriaceae and Pseudomondaceae in the C group. Eubacteriaceae has been associated with obesity in obese adults (69) and youths (67); however, increases in Eubacteriaceae have also been associated with exercise and a cellulose fiber diet in mice (70), which could have contributed to its increase in the C group. Pseudomondaceae was found to be associated with obesity and insulin resistance (IR) in humans (71), and members of this family have been speculated to have possible opportunistic pathobiont capacity to induce the inflammatory pathways associated with IR.

It is recognized that diet is a driving factor of microbial community composition in the animal kingdom (72). Therefore, one of the big advantages of equine studies is that tight regulation and uniform nature of diet can be achieved. In our study, this is reflected in the generalized similarities in the taxonomy of the bacterial communities in the treatment and control groups as revealed by 16S rRNA sequencing. There have been a number of studies investigating the effect of diet (73–75) and the effects of dietary restriction on gut microbiota in equids (52, 53). The core microbiota of horses has been shown to be relatively stable with 65% of the microbial community retained when horses were on a restricted fiber-based diet (53). Similarly, humans have a recognizable core microbiome (76) that also remains quite stable over time (58). In this study, similar to other longitudinal studies (53, 77), the PCA graph clustering shows similarities in individual horse samples over the three time points in the T group, which highlights the need for longitudinal studies, whereby the participants can act as their own controls.

In humans, high-level microbial diversity in the gut is frequently regarded as desirable, and it has been proposed that obese and overweight phenotypes are associated with decreased intestinal diversity (78). However, a recent systematic review could not find enough consistent results to corroborate these findings (16). Similarly, dietary restriction in humans has provided variable results with respect to the fecal microbiota. In a study of 49 obese/overweight individuals, dietary restriction resulted in improved gene richness and clinical phenotype (79), while a low calorie diet induced no change in the phylogenetic make up or alpha diversity of the fecal microbiota (80). In horses, an increase in diversity related to obesity has been documented (18, 52) with a decrease in diversity associated with weight loss as result of a more stringent (1% BW in DMI) restricted diet (52). In this study, the treatment group showed a significant increase in alpha diversity over time, while no change was seen in the control group. Therefore, it would be reasonable to propose that, in the treatment group, exercise may have improved the overall intestinal health of the horses, while also contributing to both weight loss and reduced waist circumference.

The benefits of physical exercise are well-documented and have been proven in humans (81). There is growing evidence that this benefit may be associated with modulation of the microbiome, with exercise modifying the microbiota in diabetic mice (82) as well as obese rats (62) and even attenuating intestinal dysbiosis in mice (61). Similarly, in humans, exercise is citied as a possible therapy for gut dysbiosis-associated disease (83). However, the effects of exercise are inconsistent. In a study of 90 overweight/obese individuals in Ireland, a moderate intensity exercise program had minimal effect on the composition or function of the fecal microbiota (47). However, a study of sedentary overweight women saw effects in half the treatment group (84). In a study comparing lean to obese adults, the effect of exercise was transient and reversible (85). To date, there is limited evidence on the effects of exercise on the equine intestinal microbiota, with this being the first study done on sedentary overweight horses. However, exercise have been associated with improvement in insulin sensitivity in overweight horses (20, 43), and perhaps, the increase in diversity of our exercised horses could be related to improve insulin dynamics. This study did not measure insulin-related parameters, and so, further studies would be required to investigate whether this is the case.

The metabolome analysis allows investigation of the functional changes associated with bacterial community and the potential changes that a weight loss program may have not only on the microbiota composition but also the associated metabolome. Although we did not observe a significant difference between the treatment and the control groups, both groups showed significant changes in metabolomic profile between the start and end of study period. This is most likely attributable to adjustment to the fiber-based diet over time.

To get a more nuanced view of microbe–metabolite–host interactions, this study used network analysis of correlations between the metabolites and OTUs found. The family Rikenellaceae was associated with urocanic acid at TP 3 in the T group. There were three other OTUs, which the analysis showed as linked to specific metabolites found, the most interesting being the family Ruminococcaceae, which was correlated to propionate.

Rikenellaceae is a saccharolytic bacteria that can ferment glucose to acid byproducts. In mouse studies, it has been shown to increase in obesity inducing high-fat diets and would be considered an obesity-related bacteria (70). Such high-fat diets have been shown to be associated with gut microbiota changes, increased adiposity, and metabolic syndrome accompanied by metabolic endotoxemia (86). Urocanic acid (UCA), a breakdown product of histidine, has been associated with immunomodulatory properties. Most notably, UCA has been shown to attenuate experimentally induced inflammatory bowel disease in mice and in *ex vivo* studies (87, 88).

Although Ruminococcaceae is highly specialized in the degradation and fermentation of cellulose in short-chain fatty acids (SCFAs), they are more commonly associated with butyrate production (89) rather than propionate as seen here. Ruminococcaceae has previously been reported to be present at increased abundance in obese horses (18) and notably have also been associated with a high-fat diet (HFD) in obese mice (90). Conversely, an abundance of Ruminococcaceae was associated with healthy controls when compared with horses with EMS (19). Propionate is an essential SCFA produced in the hindgut that provides up to 60% of the blood glucose in horses (91). Propionate has been shown, along with other SCFAs, to stimulate intestinal hormones, inhibit food intake, and protect against diet-induced obesity and insulin resistance (92) and has been proposed as a tool to be used in a novel approach to treating obesity (93).

In summary, the change in diversity of the samples in the T group between the start and end of the successful weight loss program is indicative of the effect of treatment on the fecal microbiota composition of horses. Moreover, the increased alpha diversity over time is linked with increase abundance of genus *Coprococcus* and class Clostridia, which are potential signposts of improved intestinal health.

### Limitations

In this study group, there was a mix of gender, breed, size, and place of origin, and although there was an acclimatization period, all of these factors can affect the fecal microbiota. The sample size was small, and further larger studies would be required to confirm the microbiota and metabolome findings. Although target weights were achieved, two of the seven horses in the treatment group were unable to participate fully in the prescribed exercise program, which may have affected the results. Further investigation into cardiovascular parameters through echocardiology as done by Heliczer (15) would have been preferable. However, the animals' excess weight made adequate visualization of the heart impossible. Although the time period of this study was short, it was similar to other weight loss investigations (41, 43, 53) and enabled the target weight loss to be achieved. The fecal microbiota has been shown to reflect the large intestinal microbiota, but, of course, does not represent changes directly (54). However, fecal sampling is readily accessible and non-invasive and, thus, is a valuable tool in investigations of disorders of the equine gut and particularly the large intestine. As this study was not carried out under experimental license, we did not take invasive samples to measure the effects of diet and exercise on changes in insulin dynamics and adipokine levels.

### Future Directions

As we reach for novel treatments to combat the ongoing upsurge of equine obesity, the manipulation of the intestinal microbiota is potentially a key target for future therapeutics. Therefore, we suggest longitudinal studies evaluating larger sample groups investigating the systemic effects of a weight loss program using a multiomic approach in tandem with investigation of insulin dynamics and adipokine levels. This approach could offer insights into the interaction between ongoing weight loss, increased insulin sensitivity, and the intestinal microbiome. Potentially, highlighting fecal biomarkers associated with weight loss resistance and insulin resistance could enable therapeutic modulation of the equine intestinal microbiota as has been shown to be effective in humans, such as diet supplementation (94) and fecal microbial transplant (95).

### Conclusions

In this study, it was shown that a controlled weight loss program using diet restriction and exercise can be successful and may contribute to an improvement in the intestinal health of the horses. The insights gained will be useful in designing further studies aimed at prevention and treatment of obesity and obese-associated disease in horses. With the overarching aim of the research to unravel the connections of a range of chronic inflammatory conditions linked to gut health in the horse, and, in line with the One Health paradigm, across species.

## Data Availability Statement

The data presented in the study are deposited in the ENA repository, accession number ERP127102.

## Ethics Statement

The animal study was reviewed and approved by UCD Animal Research Ethics Subcommittee AREC-E-19-26-Mulcahy. Written informed consent was obtained from the owners for the participation of their animals in this study.

## Author Contributions

NW participated in the study design, execution, data analysis, and interpretation and preparation of the manuscript. RC participated in the study design and execution of the study. VG participated in the study design and execution of the study. VD and AP participated in the study design, performed the ultrasound examination of the horses, and preparation of the manuscript. GM participated in the study design, execution, data analysis and interpretation, preparation of the manuscript, and approved the final version of the manuscript. LB performed the metabolome analysis and contributed to the preparation of the manuscript. PC, FC and RC-R carried out the 16S rRNA gene sequence analysis, bioinformatic analysis, and contributed to the preparation of the manuscript. All authors contributed to the article and approved the submitted version.

## Conflict of Interest

The authors declare that the research was conducted in the absence of any commercial or financial relationships that could be construed as a potential conflict of interest.
