# Supplementary material for: A Multiomic Approach to Investigate the Effects of a Weight Loss Program on the Intestinal Health of Overweight Horses
Source: Front Vet Sci. 2021 Jun 18;8:668120. doi: 10.3389/fvets.2021.668120 (PMC8249564; doi:10.3389/fvets.2021.668120)
Supplement: Supplementary file 1 [file Table_1.DOCX]

**Supplementary Table 1** The percentage change in ultrasound subcutaneous fat measurements before the commencement and after the cessation of the weight loss program. The five areas examined included: retroperitoneal (ventral midline), top of the crest of the neck, withers, ribs at 14^th^ intercostal space (ICS) and the tailhead. Images for each area were obtained in triplicate and averaged for statistical analysis.

US – ultrasound, ICS intercoastal space, AVG – average, NT – not taken. Minus figures indicate increase in fat depth

| Subcutaneous Fat Thickness US measurements -percentage change over 6 weeks | | | | | |
| --- | --- | --- | --- | --- | --- |
|  |  |  |  |  |  |
| Horse | **Top of Neck Crest** | **Withers** | **Rib (14^th^ ICS)** | **Tailhead** | **Retroperitoneal (Ventral midline)** |
|  |  |  |  |  |  |
|  | % | % | % | % | % |
| Control Group | | | | | |
| 1 | +0.05 | NT | -0.11 | -0.63 | -0.11 |
| 2 | -0.10 | -0.34 | -0.47 | -0.52 | +0.12 |
| 3 | +0.03 | +0.01 | -0.14 | NT | -0.23 |
| 4 | NT | NT | NT | NT | NT |
| 6 | +0.28 | +0.23 | +0.13 | NT | NT |
| 10 | +0.34 | -0.50 | +0.02 | -0.23 | -0.43 |
| 13 | -0.06 | -0.37 | -0.23 | -0.37 | -0.28 |
| Treatment Group | | | | | |
| 5 | +0.01 | -0.25 | -0.11 | -0.57 | +0.01 |
| 7 | NT | +0.52 | NT | NT | NT |
| 8 | +0.39 | +0.05 | -0.39 | -0.30 | -0.29 |
| 9 | +0.06 | -0.47 | -0.31 | -0.50 | -0.14 |
| 11 | +0.23 | -0.33 | -0.17 | -0.20 | +0.28 |
| 12 | +0.19 | -0.32 | +0.36 | NT | -0.22 |
| 14 | -0.02 | +0.53 | -0.41 | -0.00 | -0.31 |

**Supplementary Figures**

**Figure S1**

PCA analysis of the individual samples from each horse in the treatment group at each timepoint. Clustering in accordance with individual variation is evident.

**Figure S2**

A representative NMR spectrum of faecal water metabolites. Assignment of some major peaks are highlighted.
