# Supplementary figures and images for: A Multiomic Approach to Investigate the Effects of a Weight Loss Program on the Intestinal Health of Overweight Horses
Source: Front Vet Sci. 2021 Jun 18;8:668120. doi: 10.3389/fvets.2021.668120 (PMC8249564; doi:10.3389/fvets.2021.668120)

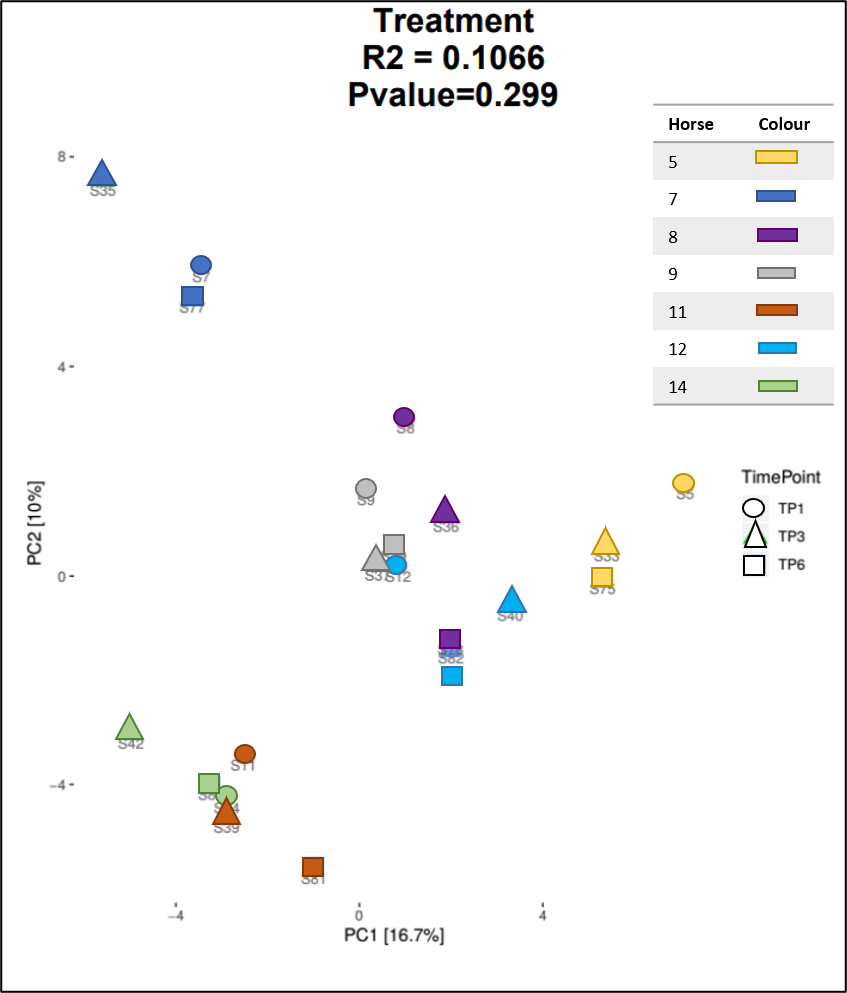

Supplement: Supplementary file 2 [file Data_Sheet_1.ZIP › Supplmentary Figures/Figure S1 PCA treatment.tif]

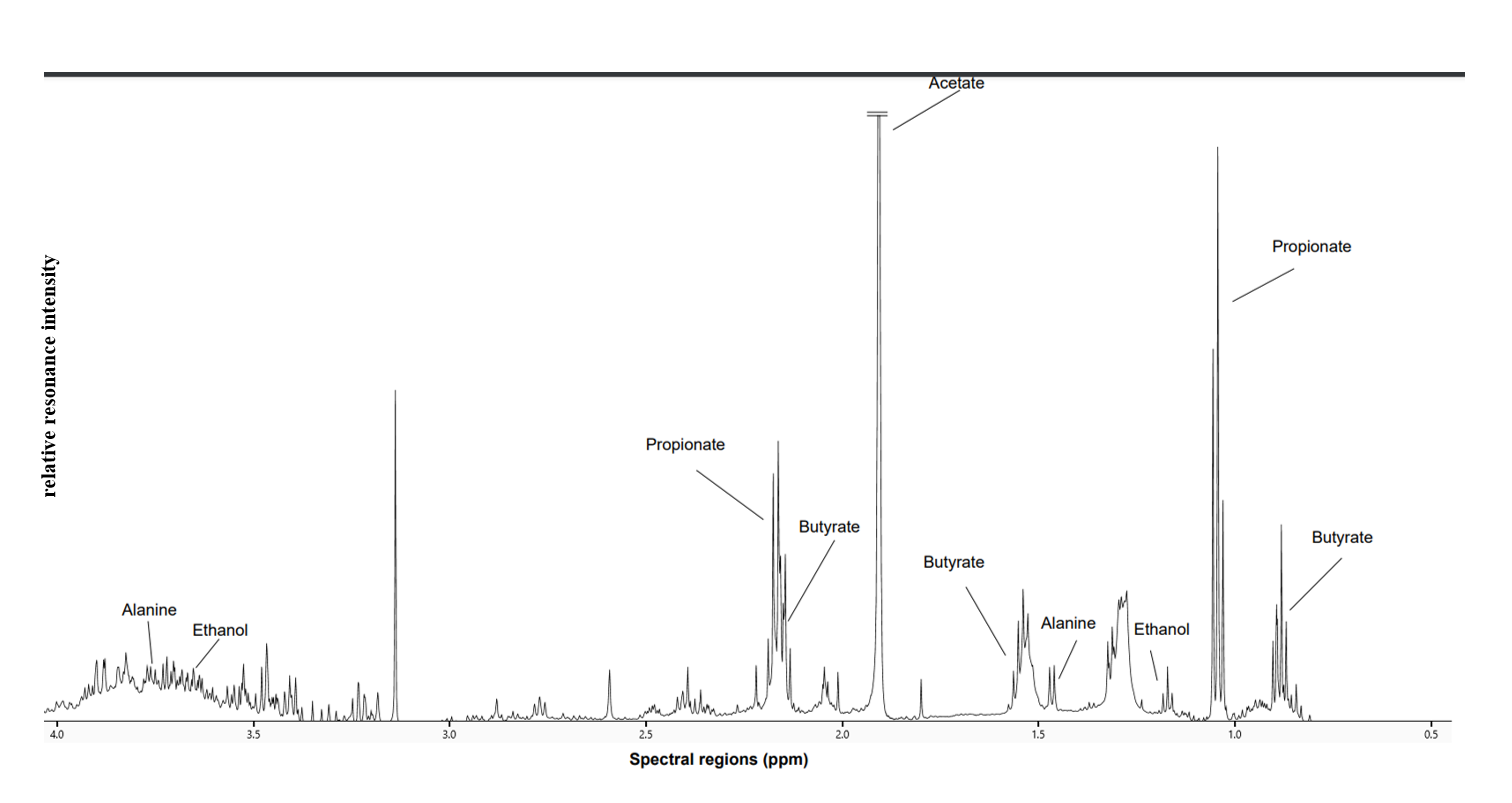

Supplement: Supplementary file 2 [file Data_Sheet_1.ZIP › Supplmentary Figures/Figure S2 NMR spectrum.tif]
